# Supplementary material for: Predicting visual function by interpreting a neuronal wiring diagram
Source: Nature. 2024 Oct 2;634(8032):113–23. doi: 10.1038/s41586-024-07953-5 (PMC11446822; doi:10.1038/s41586-024-07953-5)
Supplement: Supplementary file 6 — CRF and ERF predictions for individual TmY4 and TmY9 cells. Analogous to Supplementary Data 3, but for TmY target types. Shown are the top four monosynaptic pathways, the strongest pathway passing through each of the top ten intermediary types (ranking from Extended Data Fig. 7), and the trisynaptic pathway Tm1–TmY–Dm3–TmY (see the section entitled Prediction of spatial normalization). [file 41586_2024_7953_MOESM6_ESM.zip › DataS4/TmY9qΓèÑ/720575940620927982.pdf]

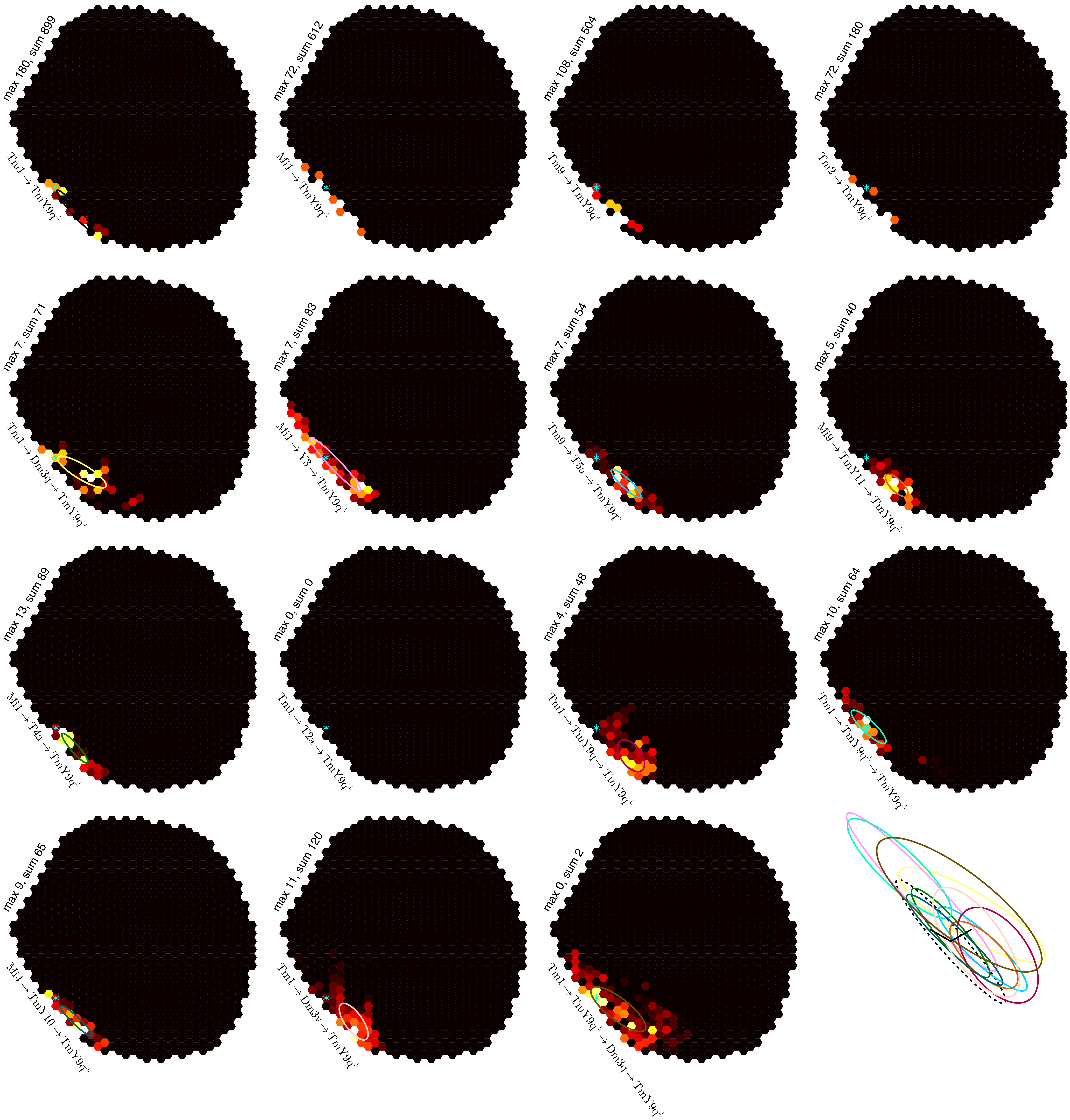
max 180, sum 899
Tm1 → TmY9q$^{\perp}$
max 72, sum 612
Mi1 → TmY9q$^{\perp}$
max 108, sum 504
Tm9 → TmY9q$^{\perp}$
max 72, sum 180
Tm2 → TmY9q$^{\perp}$
max 7, sum 71
Tm1 → Dm3q → TmY9q$^{\perp}$
max 7, sum 83
Mi1 → Y3 → TmY9q$^{\perp}$
max 7, sum 54
Tm9 → T5a → TmY9q$^{\perp}$
max 5, sum 40
Mi9 → TmY11 → TmY9q$^{\perp}$
max 13, sum 89
Mi1 → T4a → TmY9q$^{\perp}$
max 0, sum 0
Tm1 → T2a → TmY9q$^{\perp}$
max 4, sum 48
Tm1 → TmY9q → TmY9q$^{\perp}$
max 10, sum 64
Tm1 → TmY9q$^{\perp}$ → TmY9q$^{\perp}$
max 9, sum 65
Mi4 → TmY10 → TmY9q$^{\perp}$
max 11, sum 120
Tm1 → Dm3v → TmY9q$^{\perp}$
max 0, sum 2
Tm1 → TmY9q$^{\perp}$ → Dm3q → TmY9q$^{\perp}$
